# Bran Baths

**Published:** 1885-02

**Authors:** 


*Bran Baths.*—Pape obtained two brilliant results prescribing tepid bran baths two to three times a day in cases of psoriaris idiopathica. Although this treatment does not always prevent return of the disease, yet it ought to be preferred so as to rapidly remove the affection.—*Med. Chir. Rundschau.*—*La Salute.*

---

*Honor.*—Professor Guido Baccelli, ex-Minister of Public Instruction of the Kingdom of Italy, and Director of the Medical Clinic in the University of Rome, has been nominated a member of the Academy of Medicine of Berlin.

---

The University of Vienna has a library which can contain a million of books. At present there are 725,000 volumes. The reading-room has 280 numbered chairs; 120 for law students, 80 for medical students, and 80 for the students of philosophy.

